# Effects of Verteporfin on Interstitial Fluid Flow-Induced Fibrotic Transdifferentiation of Human Tenon Fibroblasts

**DOI:** 10.1167/iovs.66.4.17

**Published:** 2025-04-08

**Authors:** Janne Frömmichen, Emma Bungert, Jeanne Ströble, Moritz Gläser, Charlotte Gottwald, Kosovare Zeqiri, Thomas Reinhard, Jan Lübke, Günther Schlunck, Cornelius Jakob Wiedenmann

**Affiliations:** 1 Eye Center, Medical Center, Faculty of Medicine, University of Freiburg, Freiburg, Germany

**Keywords:** fibrosis, glaucoma, surgery, mechanotransduction, verteporfin, YAP/TAZ, fibronectin

## Abstract

**Purpose:**

Postoperative scarring remains the major challenge in achieving long-term success after glaucoma filtration surgery. In a previous study, we showed that slow continuous fluid flow is sufficient to induce fibrotic responses in human tenon fibroblasts (HTFs) in two-dimensional (2D) and three-dimensional (3D) in vitro models. In the present study, we investigated the role of the mechanosensitive Yes-associated protein (YAP) and transcriptional coactivator (TAZ) signaling pathway in flow-induced fibrosis.

**Methods:**

HTFs were exposed to continuous fluid flow for 48 or 72 hours in the presence or absence of the YAP/TAZ-transcriptional enhanced associated domain inhibitor verteporfin (VP). In a 2D model, the F-actin cytoskeleton, fibronectin 1 (FN1), YAP, and TAZ were visualized by confocal immunofluorescence microscopy. In a 3D model, mRNA was extracted, and the expression of fibrosis-associated genes was detected by quantitative PCR.

**Results:**

HTFs exposed to slow fluid flow showed increased staining intensities for YAP/TAZ. Inhibition of YAP/TAZ by VP slightly reduced flow-induced fibrotic changes in the 2D model. The flow-induced increase in the expression of the extracellular matrix (ECM) genes *COL1A1*, *CTGF*, and *FN1* was significantly inhibited by VP in the 3D model.

**Conclusions:**

Slow interstitial fluid flow activates the YAP/TAZ pathway. VP exerts antifibrotic potential by reducing morphologic changes and suppressing the expression of ECM genes induced by flow. Therefore, YAP/TAZ inhibition may exhibit therapeutic potential after glaucoma filtration surgery by inhibiting fibrotic changes induced by mechanical stimuli.

Glaucoma is a group of optic neuropathies characterized by progressive, irreversible degeneration of retinal ganglion cells.^1^ With a global prevalence of around 3.5% in a population aged between 40 and 80 years and an increasing incidence, more than 70 million people currently have glaucoma.^2^^–^^4^ This makes glaucoma, after cataracts, the second most common risk factor for vision loss and the leading cause of irreversible blindness worldwide.^5^^–^^7^ Lowering intraocular pressure (IOP) is the most effective and widely used treatment option to prevent progression.^8^^,^^9^ First introduced in 1968,^10^ trabeculectomy continues to serve as a gold standard for IOP-lowering filtration surgery. Despite improvements in surgical procedures and the use of antifibrotics such as mitomycin C and 5-fluorouracil, postoperative scarring remains the primary cause of therapeutic failure.^11^^,^^12^

In a preceding publication, we reported that slow interstitial fluid flow, as it occurs after glaucoma filtration surgery, promotes fibrosis acting on human tenon fibroblasts (HTFs). It elicited changes in cell shape, the F-actin cytoskeleton, and the deposition of fibronectin 1 (FN1) and activated the intracellular TGF-β signaling pathway to induce expression of fibrosis-related genes, such as *CTGF*, *FN1*, and *COL1A1*. Inhibition with a TGF-β receptor inhibitor (ALK5 inhibitor) reduced the effects of flow on the F-actin cytoskeleton and fibronectin 1 (FN1) bundle formation significantly in a two-dimensional (2D) model. In a three-dimensional (3D) model, the flow-induced increase in expression of fibrosis-related genes, such as *TGFB1*, *FN1*, and *COL1A1*, was significantly reduced. Western blot analysis revealed a loss of SMAD2 phosphorylation in the presence of an ALK5 inhibitor. However, testing of a TGF-β–binding antibody showed no specific effect, suggesting the involvement of additional pathways in flow-induced fibrosis.^13^ We concluded that even though slow fluid flow was shown to activate the TGF-β–Smad signaling pathway, further mechanisms like flow-dependent shear stress activation of mechanosensitive signaling pathways, such as Piezo1-signaling or the Yes-associated protein (YAP)/transcriptional coactivator (TAZ)–transcriptional enhanced associated domain (TEAD) pathway, may play a role in postoperative scarring. Hence, the model of recombinant TGF-β stimulation in a static 2D cell culture as a commonly used setup to study fibrotic processes neglects other factors such as mechanotransduction and may provide only limited insight into the scarring process after glaucoma filtration surgery.

In general, cells possess the ability to convert mechanical stimuli into biochemical signals through a mechanism known as mechanotransduction.^14^ Mechanical strain is sufficient in inducing transdifferentiation of a fibroblast into a myofibroblast, a crucial process in the formation of fibrosis.^15^ Therefore, the inhibition of downstream molecules in mechanotransduction appears to be an additional viable option for preventing scarring after glaucoma filtration surgery and warrants further investigation.

Important nuclear mediators of mechanical signals include YAP and TAZ as key components of the Hippo pathway.^16^^–^^18^ The nuclear localization and activity of YAP/TAZ appears to depend on cytoskeletal tension and the Rho/ROCK signaling pathway, operating independently from the Hippo pathway.^19^ In the nucleus, YAP interacts with TEAD transcription factors, mediating the expression of genes associated with the epithelial–mesenchymal transition and cell growth, such as *CTGF*.^20^^,^^21^ Additionally, in a cellular model of conjunctival fibrosis, YAP/TAZ were found to be central components in the TGF-β2 signaling pathway.^22^ Inhibition by verteporfin (VP) seemed to suppress the TGF-β2–induced profibrotic effect. While direct inhibition of TGF-β2 with CAT-152 failed to improve trabeculectomy outcomes,^23^ the role of YAP/TAZ activation in flow-induced fibrosis remains unclear. Given VP's antifibrotic properties,^24^^–^^27^ we investigated YAP/TAZ-mediated fibrosis in human tenon fibroblasts under flow conditions and VP’s potential inhibitory effects.

## Materials and Methods

### Antibodies and Reagents

The following primary antibodies were used for immunofluorescent staining and Western blot: monoclonal anti–α-smooth muscle actin (α-SMA) (A2547; Sigma-Aldrich, St. Louis, MO, USA), anti-cellular fibronectin 1 (F6140; Sigma-Aldrich), and anti-YAP/TAZ (D24E4; Cell Signaling Technologies, Denver, MA, USA). Secondary antibodies included goat anti-mouse immunoglobulin G (IgG) conjugated with fluorescein isothiocyanate (115-095-146; Jackson ImmunoResearch, Cambridgeshire, UK) or tetramethylrhodamine B isothiocyanate (TRITC) (GtxMu-003-J2RHOX; ImmunoReagents, Raleigh, NC, USA), donkey anti-rabbit IgG conjugated with Cy5 (711-175-152; Jackson Immuno Research Europe Ltd., Ely, UK), and peroxidase-conjugated goat anti-mouse and goat anti-rabbit secondary antibodies (111-035-003; Jackson ImmunoResearch). Furthermore, phalloidin-TRITC (P1951; Sigma-Aldrich) was applied. A detailed list of all antibody concentrations is shown in Table 1.

**Table 1.** Antibody Dilutions

Antigen/Phalloidin	Western Blot Concentration	Immunofluorescence Concentration
α-SMA	1:1000	
FN1	1:1000	1:400
YAP/TAZ	1:1000	1:200
Phalloidin-TRITC		1:500
Secondary antibody	1:10,000	1:250

For RNA isolation, a total RNA purification kit (RNeasy) (74104; Qiagen, Hilden, Germany) and RNase-free DNase (79254; Qiagen) were used. TaqMan Fast Advanced Master (4444557; Thermo Fisher, Waltham, MA, USA) was purchased for quantitative PCR analysis.

As a positive control for fibrosis induction, cells were treated with 1 ng/mL (final concentration) recombinant TGF-β1, obtained from Pepro-Tech (Cranbury, NJ, USA). Verteporfin (5305; Tocris, Wiesbaden-Nordenstadt, Germany) was dissolved in dimethylsulfoxide (DMSO) with a final concentration of 1 µM VP and 0.01% DMSO. All experiments using VP were carried out under the most dark conditions possible to prevent a phototoxic effect on the cells.

### Cell Culture

Primary HTFs were isolated from small tenon biopsy specimens, obtained from ophthalmologically healthy patients undergoing strabismus surgery. The tenets of the Declaration of Helsinki were followed, and an institutional ethics committee (172/18) approval was granted (July 13, 2018). Cells in early passages were stored in liquid nitrogen in 10% DMSO in fetal bovine serum (FBS). HTFs between passages 3 and 8 were used for all experiments. By default, cells were maintained in low glucose (1 g/L D-glucose) Dulbecco's modified Eagle medium (DMEM) (11880-63; Life Technologies, Carlsbad, CA, USA) supplemented with 10% heat-inactivated fetal bovine serum (10270106; Life Technologies), 100 U/mL penicillin, and 100 µg/mL streptomycin (P4333; Sigma-Aldrich) and 2 mM L-glutamine (25030-081; Life Technologies) under standard conditions (37°C, 5% CO_2_, and 90% humidity). All experiments were performed at least three times.

For static conditions, 150,000 HTFs were seeded in 60-mm Petri dishes and initially incubated for 24 hours in 0.2% FBS medium. One hour before stimulation of the positive controls with 1 ng/mL TGF-β1 and VP, medium containing 1 µM VP was added to the defined Petri dishes without additionally dissolved TGF-β1. The medium was changed every 24 hours. After 72 hours, RNA and protein samples were obtained from the Petri dishes. For each condition, two Petri dishes were used as biological replicates.

### Fluid Flow in the 2D Setting

**Figure 1.**
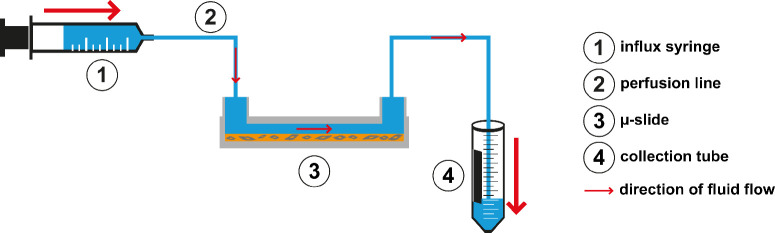
The 2D setup. Flow was induced by an influx syringe (1) programmed to a flow rate of 150 µL/h. The µ-slide (3) inlet and outlet port were connected to perfusion lines (2). The outlet of the µ-slide led into a collection tube (4). (Figure modified from Wiedenmann et al.^13^)

For stimulation with a slow interstitial fluid flow in a 2D cell culture setup, 15,000 HTF cells were seeded in µ-slides I Luer 0.8 mm (80196; ibidi GmbH, Gräfelfing, Germany), cultured in medium containing 0.2% FBS, and incubated for 24 hours (Fig. 1). Before starting the flow stimulation, the medium was changed to the respective test (1 µM VP) or control media. The µ-slides were then connected to a pump via infusion tubes and perfused with medium containing 0.2% FBS at a flow rate of 150 µL/h for 48 hours, resulting in a shear stress of approximately 0.0006246 dyn/cm^2^ as specified in the µ-slides. Within each experiment, there were corresponding static controls where medium was changed every 24 hours. For each condition, two µ-slides were used as biological replicates.

### Interstitial Fluid Flow in the 3D Setting

**Figure 2.**
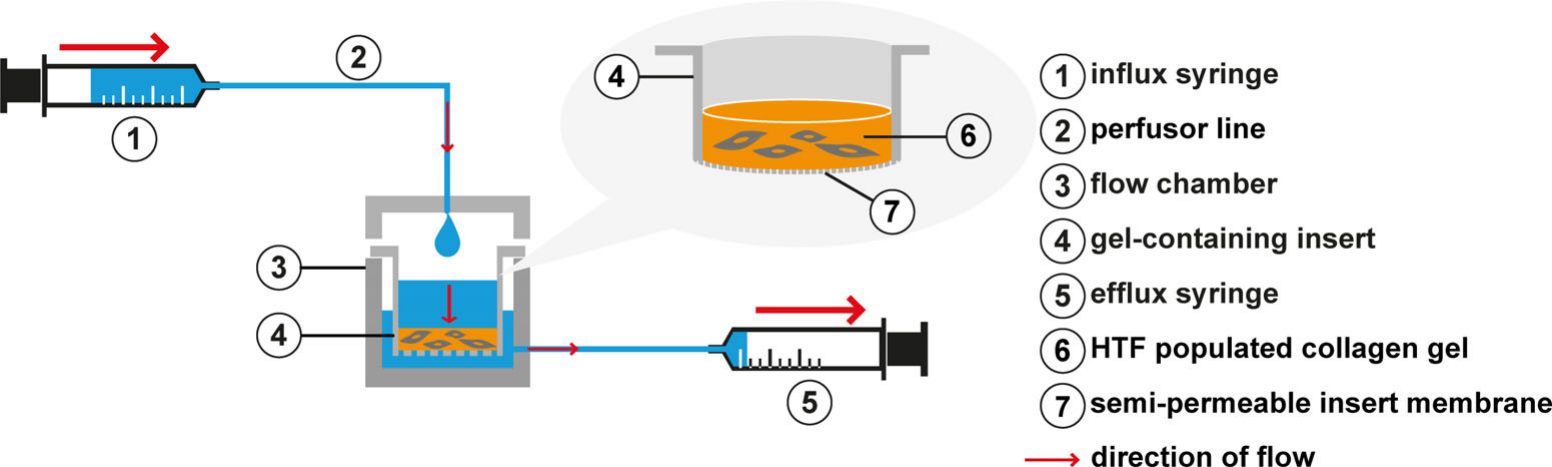
The 3D setup. Flow was induced by an influx syringe (1) programmed to a flow rate of 180 µL/h. The custom-made 3D flow chamber (3) was connected to the influx syringe (1) by a perfusor line (2) attached to the chamber lid. Following the hydrostatic pressure gradient, flow was generated through the cell-populated collagen gel (6) of the insert (4) and the semipermeable insert membrane bottom (7) into the outer chamber (3). To maintain a hydrostatic gradient, the outer chamber (3) was drained close to the bottom by an efflux syringe (5) aspirating at the same flow rate as the influx syringe. (Figure modified from Wiedenmann et al.^13^)

For the 3D approach, HTFs were embedded in a collagen gel suspension culture (Fig. 2). For each collagen gel, 30,000 cells, dissolved in 50 µL medium (0.4% FBS), 400 mL PureCol Type I Bovine Collagen Solution (3 mg/mL), 50 µL DMEM (10×), 32.5 µL NaHCO_3_ (7%), 10 µL HEPES (1 M), and 4 µL NaOH (1 M), were required, resulting in a final collagen concentration of 2.18 mg/mL (#5005; Advanced BioMatrix, Carlsbad, CA, USA). The mixture was transferred into an insert for 12-well plates with a pore diameter of 3 µm (Thincert cell culture insert; Greiner Bio-One, 665630, Frickenhausen, Germany) and incubated at 37°C for collagen polymerization. After 1 hour, 1 mL of medium (1% FBS) was added to the insert, and 2 mL of medium was added around the insert. The gels were cultivated statically for 3 days, with a medium change on the second day. After 3 days of incubation, the inserts were transferred to a flow chamber, and the medium was changed for the respective treatment (1 µM VP) or control media. The cells were exposed to a continuous fluid flow of 180 µL/h for 3 days (72 hours) by an influx syringe (filled with respective medium containing 1% FBS) and an efflux syringe, leading to a maximum shear stress of 0.066 dyn/cm^2^. The cultures and the flow chamber were maintained in a humidified 37°C, 5% CO_2_ incubator. Gels in inserts in a 12-well plate served as static controls. For each condition, two µ-slides were used as biological replicates.

### Confocal Immunofluorescence Microscopy

Confocal immunofluorescence microscopy was used to assess morphologic changes, deposition of extracellular matrix proteins, and changes in the intracellular distribution of proteins induced by fluid flow and corresponding treatment substances. After stopping the flow, cells were directly fixed with 4% paraformaldehyde for 20 minutes and permeabilized with 0.1% Triton X-100 (SLBV4122; Sigma-Aldrich). Subsequently, they were blocked for 1 hour with blocking solution (Dulbecco's phosphate-buffered saline [DPBS] [1×] + Triton X-100 [0.1%]) + normal goat serum (NGS) (5%; Dianova, Hamburg, Germany) and afterward incubated with primary antibodies against FN1 or YAP/TAZ in 1% NGS and 0.1% Triton X-100 in DPBS overnight at 4°C. The µ-slides were then washed again with 0.1% Triton X-100, and the secondary antibody, including Phalloidin-TRITC, dissolved in the antibody solution, was applied. To stain the cells embedded in collagen gels, the gels were washed with DPBS (1×) on a shaker and solutions contained 0.5% Triton X-100. Stains were visualized with a laser scanning confocal microscope (TCS SP8; Leica Microsystems, Bensheim, Germany), and z-stacks were scanned with z-position increments of 1 µm (µ-slides) or 2 µm (3D gels). All images within an experiment were acquired using identical laser settings. Signal intensities for the proteins of interest were quantified using ImageJ 1.53a (Rasband, W.S.; National Institutes of Health, Bethesda, MD, USA).

### Gene Expression Analysis

Collagen gels from 3D cultures were transferred to Eppendorf tubes, washed with DPBS at 37°C on a shaker (900 rotations per minute), and then digested with 2 mg/mL Collagenase D (11088858001; Roche AG, Basel, Switzerland) for 40 minutes. Collagenase digestion was stopped using 70 µL EDTA (10 mM). Cells were spun down at 500 *g* and 4°C for 10 minutes, and the cell pellet was lysed using a RNeasy Mini Kit and QIAshredder homogenizer columns (Qiagen) according to the manufacturer's instructions. Samples were directly transcribed into cDNA or stored at −80°C. The amount of RNA was measured using a NanoDrop-1000 Spectrophotometer (ND-1000; PeqLab Biotechnologie GmbH, Erlangen, Germany). Reverse transcription to first-strand cDNA was carried out by SuperScript IV Reverse-Transcriptase (18090010; Thermo Fisher Scientific) at 52°C for 10 minutes and subsequent separation of the RNA and cDNA strands from each other required another 10 minutes at 80°C. The samples were then stored at −20°C.

Gene expression analysis was performed using real-time quantitative PCR (qPCR) using specific TaqMan sets of primers (Table 2) and probes coupled to FAM (6-carboxyfluorescein) as listed in (Table 2). Three duplicates of the samples, as well as biological replicates, were pipetted in a 96-well microtiter plate (LightCycler 480 Multiwell Plate 96, white; Roche AG), capped with ultraclear Optical Flat 8-Cap Strips (TCS 0803; Bio-Rad, Feldkirchen, Germany), and amplified using TaqMan Universal PCR Master Mix (4364340; Thermo Fisher Scientific). The cycling program (run on a LightCycler 96; Roche AG) comprised a 25-second initial denaturation at 95°C, followed by 40 recurring cycles of 3 seconds of denaturation at 95°C and 30 seconds of hybridization and primer elongation at 60°C. Ct values (threshold cycle) were estimated by LightCycler 96 Software Version 1.1.0.1320 (Roche) and analyzed using the 2^–^^ΔΔCt^ method.^28^ The Ct values of each triplet were averaged and subtracted from those of the housekeeping gene β2-microglobulin (B2M) for relative quantification. The result of the analysis is the fold change/multiple of mRNA expression under stimulation relative to the control.

**Table 2.** TaqMan Primers and Probe Sets Used for qPCR

Gene	Primer	Company
ACTA2	Hs00426835_g1	Thermo-Fisher
B2M	Hs00187842_m1	
COL1A1	Hs00164004_m1	
CTGF	Hs00170014_m1	
FN1	Hs01549976_m1	
TGFB1	Hs00998133_m1	

### Western Blot

HTFs cultured on Petri dishes were placed on ice, washed with PBS, and treated with 150 µL T-PER buffer (243205; Sigma-Aldrich) with added protease and phosphatase inhibitors (04906845001 and 04693159001; Roche). The lysate was then collected into microcentrifuge tubes using a cell scraper and subsequently centrifuged for 10 minutes at 14,000 *g*. Afterward, a BCA-Essay was performed using the Pierce BCA Protein Assay Kit (23227; Thermo Fischer Scientific). For Western blotting, the supernatant containing the protein extracts was boiled after adding one-fourth sample volume of a 4× Laemmli sample buffer (1610747; Bio-Rad) with 10% β-mercaptoethanol (BCBQ7289V; Sigma-Aldrich) and subjected to SDS–polyacrylamide gel electrophoresis. Proteins were transferred onto polyvinylidene difluoride membranes. Membranes were activated in methanol, blocked in 3% BSA in TBST (pH 7.4, 0.1% Tween 20) for 30 minutes and incubated with primary antibody overnight at 4°C and a peroxidase-conjugated secondary antibody for 60 minutes at room temperature. After each incubation step, membranes were washed in TBST four times for 10 minutes, respectively. Peroxidase was visualized by enhanced chemiluminescence (Pierce ECL Plus Western Blotting Substrate; Thermo Fisher Scientific), and signal intensities were measured using a Fusion FX system (Vilber, Marne-la-Valée, France) at appropriate times.

### Statistical Analysis

Statistical analyses were carried out using the R software version 4.0.1 (R Project for Statistical Computing, Vienna, Austria). Simultaneous tests for general linear hypotheses were performed by “glht” (multcomp). For analysis of variance, we used “aov” (stats). For multiple comparisons of means, we used Dunnett contrasts. *P* values below 0.05 were considered statistically significant.

## Results

### Effects of VP on TGF-β1–Stimulated HTFs Under Static Conditions

**Figure 3.**
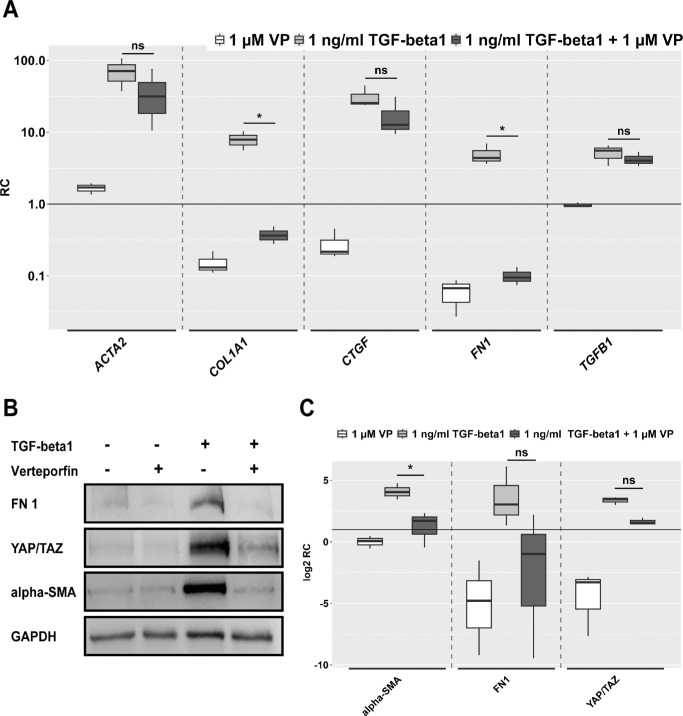
Effect of 1 µM VP on HTFs under static conditions with and without TGF-β1 (1 ng/mL). Cells were preincubated in Petri dishes in starvation medium (0.2% FBS) and treated with respective conditions for 48 hours. (**A**) qPCR shows relative expression of the fibrosis-associated genes alpha smooth muscle actin (*ACTA2*), collagen type I alpha 1 chain (*COL1A1*), connective tissue growth factor (*CTGF*), fibronectin 1 (*FN1*), and *TGFB1. n* = 3. (**B**) Western blot for protein levels of FN1, YAP, TAZ, α-SMA, and GAPDH. (**C**) Semiquantitative summary representation in mean signal intensity of Western blots for FN1, YAP/TAZ, and α-SMA, normalized to glyceraldehyde-3-phosphate dehydrogenase (GAPDH) of three independent experiments. The relative change (RC) is represented logarithmically, and the control group is represented by a horizontal line at y = 1. *Asterisks* indicate significance in Dunnett's *t*-test comparing the TGF-β1–treated condition with the TGF-β1 plus VP condition (ns *P* > 0.05, *P < 0.05).

The effect of 1 µM VP without light activation was tested under static conditions on HTFs treated with 1 ng/mL recombinant TGF-β1 as a positive control (Fig. 3). HTFs were cultured in Petri dishes and stimulated with the respective medium for 3 days. This was followed by RNA isolation for qPCR or protein harvesting for Western blotting. Analysis of the qPCR data (A) showed a strong increase in gene expression of *ACTA2*, *COL1A1*, *CTGF*, *FN1*, and *TGFB1* when HTFs were stimulated with 1 ng/mL TGF-β1. In addition, 1 µM VP significantly inhibited the increased expression of *COL1A1* and *FN1* under TGF-β1 stimulation below the control level of 1. The TGF-β1–induced increase in expression of *ACTA2* and *CTGF* was weakly but not significantly reduced by VP, whereas VP did not appear to have any effect on *TGFB1* expression. Western blot analysis (B) showed an increase in FN1, YAP/TAZ, and α-SMA concentrations after TGF-β1 stimulation. In total, 1 µM VP was sufficient to maintain FN1 and α-SMA levels at the control baseline and reduced the YAP/TAZ concentration after TGF-β1 stimulation.

### Flow-Induced Morphologic Effects on F-actin Are Reduced by the YAP/TAZ Inhibitor VP in the 2D Model

**Figure 4.**
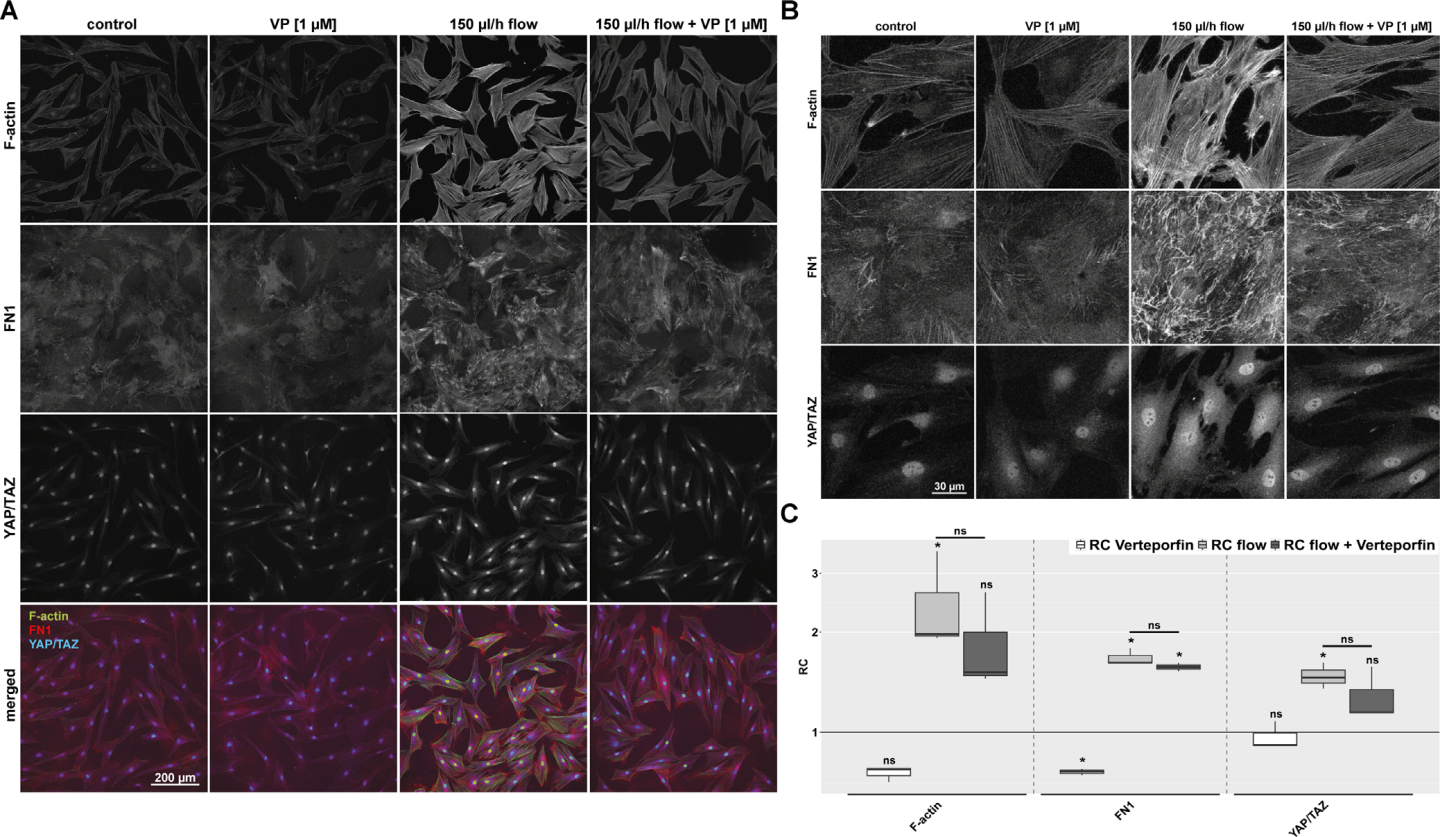
Effect of VP on F-actin, FN1, and YAP/TAZ localization under static and flow conditions. (**A**) Immunofluorescence images of F-actin, FN1, and YAP/TAZ localization under static and flow conditions (150 µL/h, 48 hours) either with or without 1 µM VP for 2 days. All experiments with 1 µM VP were conducted in darkness. Images were acquired with identical image acquisition and representation settings for all conditions throughout the experiment. Cell count for the shown image: no flow control, 56 cells; no flow and VP, 60 cells; flow, 57 cells; and flow and VP, 56 cells. (**B**) Representative high-magnification fluorescence images captured from the same experimental setup as shown in (**A**). *n* = 3. (**C**) Relative change (RC) in mean signal intensity of F-actin, FN1, and total YAP/TAZ in the confocal images from the three different experiments as in (**A**) and (**B**). *Asterisks* indicate significance in Dunnett's *t*-test (ns *P* > 0.05, *P < 0.05).

The effect of 1 µM VP without light activation on HTFs was tested under static and flow conditions in the 2D model (Fig. 4). As previously described in Wiedenmann et al.,^13^ untreated HTFs showed an elongated morphology with few, thin, and weakly stained F-actin fibers. The distribution of cellular FN1 was diffuse and did not exhibit relevant bundle formation. YAP/TAZ staining was predominantly nuclear, with a low cytosolic signal also detected. HTFs treated with VP showed a similar distribution pattern for F-actin, FN1, and total YAP/TAZ, but signal intensity for these proteins was slightly reduced in the presence of VP. In comparison, the morphology of HTFs exposed to 150 µL/h fluid flow was less elongated and more compact. The number and thickness of F-actin stress fibers appeared to increase. Additionally, the signal intensity for FN1 was increased with a more arranged appearance aligned with the actin-stress fibers. Both cytosolic and nuclear YAP/TAZ staining increased significantly, but no shift from the cytosol to the nucleus was observed. F-actin stress fibers, FN1, and YAP/TAZ intensity were all strongly upregulated under flow. The addition of the YAP/TAZ inhibitor VP showed a trend to diminish the flow-induced fibrotic changes of F-actin, FN1, and YAP/TAZ.

### YAP/TAZ Inhibition by VP Reduces the Expression of the Extracellular Matrix Genes *COL1A1*, *CTGF*, and *FN1* and the Staining Intensity for F-actin Under Flow Conditions in the 3D Model

**Figure 5.**
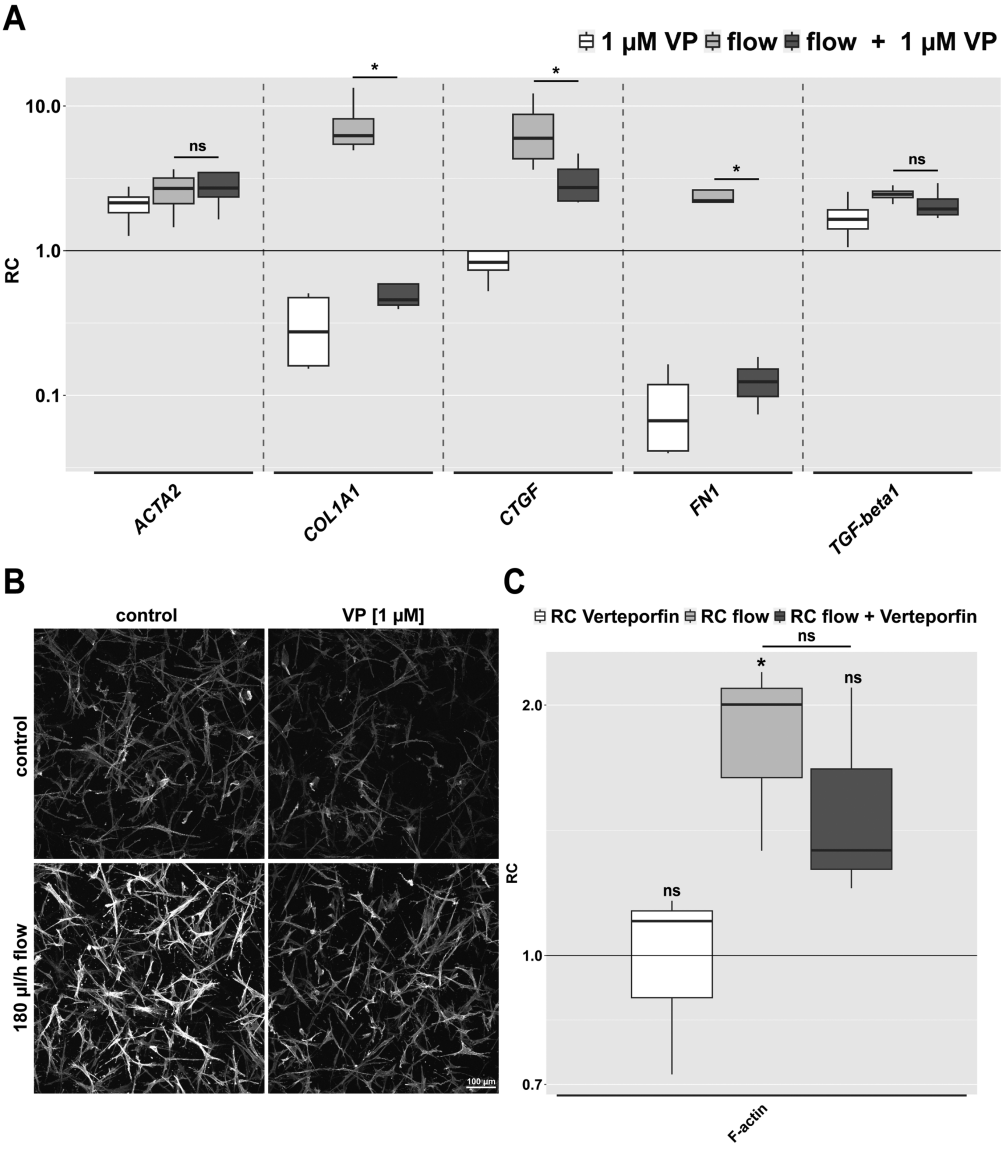
Effect of VP on the relative expression of the fibrosis-associated genes *ACTA2*, *COL1A1*, *CTGF*, *FN1*, and *TGFB1* and HTF morphology under static and flow conditions in the 3D model. Samples from conditions with 180 µL/h flow stimulation for 72 hours in the absence or presence of 1 µM VP were compared to static samples. (**A**) The relative change is represented logarithmically; the control group is represented by a horizontal line at y = 1. *Asterisks* indicate significance in Dunnett's *t*-test comparing flow to flow plus VP (ns *P* > 0.05, *P < 0.05. *n* = 4. (**B**) Confocal immunofluorescence images (z-stack projections) of F-actin under static and flow conditions (180 µL/h, 72 hours) either with or without 1 µM VP. Images were acquired with identical image acquisition and representation settings for all conditions throughout the experiment. Cell count for the shown image: no flow control, 204 cells; flow control, 164 cells; no flow and VP, 210 cells; and flow and VP, 171 cells. *n* = 3. (**C**) Relative change (RC) in mean signal intensity of F-actin in the confocal images from the three different experiments as in (**B**). *Asterisks* indicate significance in Dunnett's *t*-test (ns *P* > 0.05, *P < 0.05).

The effect of VP on the expression of selected fibrosis-associated genes was examined in the 3D model (Fig. 5A). Under static conditions, VP reduced the expression of *COL1A1* and *FN1.* However, *ACTA2* expression was slightly increased. Cells, stimulated with interstitial fluid flow and treated with 1 µM VP, showed a lower expression of *COL1A1*, *CTGF*, and *FN1* compared to the flow control group. The increase in expression of *COL1A1* and *FN1* was reduced below the control level. *CTGF* was also significantly reduced by VP but not to the expression level of the control. Gene expression of *ACTA2* and *TGFB1* under flow conditions remained unchanged by VP. Overall, VP appears to preferentially influence the expression of structural extracellular matrix (ECM) proteins, such as collagen 1 and FN1 in HTFs.

Immunofluorescence staining of HTFs embedded in collagen gels showed an increase in F-actin staining after 3 days of slow interstitial fluid flow (Fig. 5B). Treatment with 1 µM VP tended to decrease this effect. Cells treated with VP alone presented similar to their control group, indicating the absence of cytotoxic side effects under strict light restriction.

## Discussion

Postoperative scarring following glaucoma filtration surgery remains the primary obstacle to achieving long-term therapeutic success. Many different contributing factors such as cytokines and growth factors, immune cells, and the surrounding tissue are involved in the scarring process. We have recently shown that slow fluid flow alone, as it occurs after glaucoma filtration surgery, is sufficient to induce fibrotic changes in HTFs.^12^^,^^13^ The interstitial fluid flow, as we applied in our experiments, exerts shear forces of less than 0.1 dyn/cm^2^, which is several orders of magnitude below the shear forces of intravascular fluid flow (1 to 100 dyn/cm^2^).^29^ In our model, immunofluorescence staining revealed a significant enhancement of F-actin and FN1 staining intensity by flow. Additionally, slow interstitial fluid flow increased the expression of the fibrosis-associated genes *ACTA2*, *COL1A1*, *CTGF*, *FN1*, and *TGFB1*, which is in line with our previous observations. Although 2D and 3D models are not directly comparable, in both settings, a profibrotic response was induced by slow fluid flow.

In our 2D model, slow fluid flow in low serum conditions (0.2% FBS) led to an increase in staining intensity for the small nuclear molecules YAP/TAZ (Fig. 4). Considering the continuous mechanical stress that the filtering bleb undergoes after glaucoma surgery, the activation of YAP/TAZ may play a significant role in the development of fibrosis. The detailed biochemical mechanism by which shear stress activates YAP/TAZ and increases its nuclear concentration is not yet fully understood. In some cases, this mechanism is described as independent of Hippo signaling.^30^^–^^34^ The regulation of actin cytoskeletal tension via the Rho/ROCK signaling pathway has been highlighted as crucial for YAP/TAZ activation. Dupont et al.^19^ speculate that actin stress fibers inhibit a YAP/TAZ inhibitor and thus contribute to an increase in intracellular YAP/TAZ. It has also been proposed that angiomotins directly affect YAP/TAZ activity by sequestering them in the cytoplasm.^35^^,^^36^ When angiomotins interact directly with F-actin, they are prevented from binding to Yap, leading to its activation.^37^

To further investigate the possible role of YAP/TAZ in flow-induced fibrosis, we used VP, which is a clinically approved drug that has been used in photodynamic therapy for neovascular macular degeneration. It preferentially binds to the endothelium of immature blood vessels and generates reactive oxygen radicals when activated by laser light.^38^ Without light activation, VP has been identified to inhibit the YAP/TAZ-TEAD signaling pathway, exerting antiproliferative and antifibrotic effects.^24^^–^^26^ Currently, there is no authorized concentration for the subconjunctival use of VP. We initially stimulated static 2D HTF cultures with recombinant TGF-β1 to generate a strong and reproducible profibrotic effect (Fig. 3). The expression analysis of profibrotic genes by qPCR showed a significant inhibition of *COL1A1* and *FN1* expression by 1 µM VP. At the protein level, 1 µM VP maintained baseline FN1 and α-SMA and reduced YAP/TAZ levels. Thus, the selected concentration reproducibly inhibited TGF-β1–induced fibrotic changes in HTFs. Consistent with our results, an earlier in vitro study on primary human conjunctival fibroblasts treated with TGF-β2 showed profibrotic effects linked to the YAP/TAZ pathway. TGF-β2 stabilized YAP/TAZ, and its inhibition by VP exerted antifibrotic effects.^22^

While an antifibrotic effect of VP has been established in a static TGF-β–induced model, its impact on flow-induced fibrosis in HTFs has not been previously explored. Immunofluorescence staining in our 2D model showed a VP-induced reduction of the F-actin skeleton in HTFs, whereas only a weak impact on the staining intensity of FN1 and YAP/TAZ was observed. However, in each experiment, VP led to a consistent reduction in YAP/TAZ staining intensity, and the morphologic structure of deposited FN1 was altered with less bundle formation. This suggests a flow-induced upregulation of YAP/TAZ expression with little long-term effect on its nuclear/cytosolic ratio. Nakajima et al.^39^ cultured human pulmonary endothelial cells under static or flow conditions with a shear force of 15 dynes/cm² and performed an immunofluorescence analysis after 10 to 30 minutes of flow exposure, which is in contrast to our study applying a much smaller shear force of 0.0006 dynes/cm² and assessing effects after 48 hours. Rapid YAP/TAZ translocation inducing a predominantly nuclear localization appears to be an earlier event, whereas our observations reflect later time points when different equilibrium states of nucleo-cytoplasmic distribution seem to prevail. Furthermore, in the 2D model, cells were cultured on a stiff plastic surface, known to significantly enhance baseline YAP/TAZ levels,^19^ as well as nuclear YAP/TAZ localization. This is reflected in the predominantly nuclear YAP/TAZ staining observed under control conditions in our 2D model, which may diminish or mask shear-induced effects on localization. In contrast, our 3D collagen gel model provides a more physiologically relevant environment for cell culture, mimicking the softer conditions and interstitial fluid flow present in vivo. Although the formation and strengthening of the cytoskeleton is diminished in a less stiff environment,^40^^,^^41^ we consistently observed a significant increase in profibrotic gene expression. In this context, the application of VP significantly reduced the expression of *COL1A1*, *CTGF*, and *FN1*. To ensure that the effects exerted by VP were not due to cytotoxic cell disruption, we stained F-actin in static 3D control experiments. After VP incubation, the cells presented similar to untreated controls. However, a slight reduction in F-actin–stained stress fibers by 1 µM VP could be observed, consistent with a loss of mechanotransduction signals.

In line with our results, VP reduced fibrotic changes induced by other stimuli in multiple studies. In mice with unilateral ureteral obstruction, treatment with VP counteracted induced renal fibrosis.^24^ A study on dermal fibrosis in mice assessed the effect of VP on wound healing. Small incisions in the dorsal skin applied with constant tension showed less scarring with VP treatment compared to controls.^27^ In a study on trabecular meshwork (TM) cells, VP reduced TM cell–mediated collagen gel contraction in a dose-dependent manner, potentially lowering IOP by improving aqueous humor outflow.^42^

YAP/TAZ inhibition may exhibit therapeutic potential after glaucoma filtration surgery by counteracting fibrotic changes induced by mechanical stimuli and TGF-β. Despite being pharmacologically approved and clinically used for AMD treatment, the practical application of VP may be hindered by its phototoxicity when activated by light. Localization of the filtering bleb underneath the eyelid and strict light avoidance immediately after VP treatment could possibly reduce this side effect.

In conclusion, our data suggest that slow interstitial fluid flow is sufficient to activate YAP/TAZ signaling in HTFs, which appears crucial for enhanced expression of structural ECM components such as collagen 1 and fibronectin. YAP/TAZ inhibition may thus be suitable to diminish fibrotic ECM deposition following filtering glaucoma surgery and improve its long-term results.

## References

[1] Fechtner RD, Weinreb RN. Mechanisms of optic nerve damage in primary open angle glaucoma. *Surv Ophthalmol*. 1994; 39: 23–42. 7974188 10.1016/s0039-6257(05)80042-6

[2] Kapetanakis VV, Chan MPY, Foster PJ, Cook DG, Owen CG, Rudnicka AR. Global variations and time trends in the prevalence of primary open angle glaucoma (POAG): a systematic review and meta-analysis. *Br J Ophthalmol*. 2016; 100: 86–93. 26286821 10.1136/bjophthalmol-2015-307223 PMC4717368

[3] Quigley HA . Glaucoma. *Lancet*. 2011; 377: 1367–1377. 21453963 10.1016/S0140-6736(10)61423-7

[4] Tham Y-C, Li X, Wong TY, Quigley HA, Aung T, Cheng CY. Global prevalence of glaucoma and projections of glaucoma burden through 2040: a systematic review and meta-analysis. *Ophthalmology*. 2014; 121: 2081–2090. 24974815 10.1016/j.ophtha.2014.05.013

[5] Bourne RRA, Taylor HR, Flaxman SR, et al. Number of people blind or visually impaired by glaucoma worldwide and in world regions 1990 –2010: a meta-analysis. *PLoS One*. 2016; 11: e0162229. 27764086 10.1371/journal.pone.0162229 PMC5072735

[6] Steinmetz JD, Bourne RRA, Briant PS, et al. Causes of blindness and vision impairment in 2020 and trends over 30 years, and prevalence of avoidable blindness in relation to VISION 2020: the Right to Sight: an analysis for the global burden of disease study. *Lancet Global Health*. 2021; 9: e144–e160. 33275949 10.1016/S2214-109X(20)30489-7 PMC7820391

[7] Burton MJ, Ramke J, Marques AP, et al. The lancet global health commission on global eye health: vision beyond 2020. *Lancet Global Health*. 2021; 9: e489–e551. 33607016 10.1016/S2214-109X(20)30488-5 PMC7966694

[8] Leske MC, Heijl A, Hussein M, et al. Factors for glaucoma progression and the effect of treatment: the early manifest glaucoma trial. *Arch Ophthalmol*. 2003; 121: 48–56. 12523884 10.1001/archopht.121.1.48

[9] The Advanced Glaucoma Intervention Study (AGIS): 1. Study design and methods and baseline characteristics of study patients. *Controlled Clin Trials*. 1994; 15: 299–325. 7956270 10.1016/0197-2456(94)90046-9

[10] Cairns JE. Trabeculectomy: preliminary report of a new method. *Am J Ophthalmol*. 1968; 66: 673–679. 4891876

[11] Landers J, Martin K, Sarkies N, Bourne Watson R.P. A twenty-year follow-up study of trabeculectomy: risk factors and outcomes. *Ophthalmology*. 2012; 119: 694–702. 22196977 10.1016/j.ophtha.2011.09.043

[12] Schlunck G, Meyer-ter-Vehn T, Klink Grehn T,F. Conjunctival fibrosis following filtering glaucoma surgery. *Exp Eye Res*. 2016; 142: 76–82. 26675404 10.1016/j.exer.2015.03.021

[13] Wiedenmann CJ, Gottwald C, Zeqiri K, et al. Slow interstitial fluid flow activates TGF-β signaling and drives fibrotic responses in human tenon fibroblasts. *Cells*. 2023; 12: 2205. 37681937 10.3390/cells12172205 PMC10486805

[14] Ingber DE. Cellular mechanotransduction: putting all the pieces together again. *FASEB J*. 2006; 20: 811–827. 16675838 10.1096/fj.05-5424rev

[15] Hinz B, Mastrangelo D, Iselin CE, Chaponnier C, Gabbiani G. Mechanical tension controls granulation tissue contractile activity and myofibroblast differentiation. *Am J Pathol*. 2001; 159: 1009–1020. 11549593 10.1016/S0002-9440(10)61776-2 PMC1850455

[16] Kwon H, Kim J, Jho E-H. Role of the Hippo pathway and mechanisms for controlling cellular localization of YAP/TAZ. *FEBS J*. 2022; 289: 5798–5818. 34173335 10.1111/febs.16091

[17] Ma S, Meng Z, Chen Guan R,K-L. The Hippo pathway: biology and pathophysiology. *Annu Rev Biochem*. 2019; 88: 577–604. 30566373 10.1146/annurev-biochem-013118-111829

[18] Moya IM, Halder G. Hippo-YAP/TAZ signalling in organ regeneration and regenerative medicine. *Nat Rev Mol Cell Biol*. 2019; 20: 211–226. 30546055 10.1038/s41580-018-0086-y

[19] Dupont S, Morsut L, Aragona M, et al. Role of YAP/TAZ in mechanotransduction. *Nature*. 2011; 474: 179–183. 21654799 10.1038/nature10137

[20] Zhao B, Ye X, Yu J, et al. TEAD mediates YAP-dependent gene induction and growth control. *Genes Dev*. 2008; 22: 1962–1971. 18579750 10.1101/gad.1664408 PMC2492741

[21] Vassilev A, Kaneko KJ, Shu H, Zhao Y, DePamphilis ML. TEAD/TEF transcription factors utilize the activation domain of YAP65, a Src/Yes-associated protein localized in the cytoplasm. *Genes Dev**.* 2001; 15: 1229–1241. 11358867 10.1101/gad.888601 PMC313800

[22] Futakuchi A, Inoue T, Wei FY, et al. YAP/TAZ are essential for TGF-β2–mediated conjunctival fibrosis. *Invest Ophthalmol Vis Sci*. 2018; 59: 3069–3078. 30025139 10.1167/iovs.18-24258

[23] Khaw P, Grehn F, Hollo G, et al. A phase III study of subconjunctival human anti–transforming growth factor β2 monoclonal antibody (CAT-152) to prevent scarring after first-time trabeculectomy. *Ophthalmology*. 2007; 114: 1822–1830.e2, doi:10.1016/j.ophtha.2007.03.050. 17908591

[24] Szeto SG, Narimatsu M, Lu M, et al. YAP/TAZ are mechanoregulators of TGF-β-Smad signaling and renal fibrogenesis. *J Am Soc Nephrol*. 2016; 27: 3117–3128. 26961347 10.1681/ASN.2015050499 PMC5042658

[25] Liu-Chittenden Y, Huang B, Shim JS, et al. Genetic and pharmacological disruption of the TEAD–YAP complex suppresses the oncogenic activity of YAP. *Genes Dev*. 2012; 26: 1300–1305. 22677547 10.1101/gad.192856.112 PMC3387657

[26] Brodowska K, Al-Moujahed A, Marmalidou A, et al. The clinically used photosensitizer Verteporfin (VP) inhibits YAP-TEAD and human retinoblastoma cell growth in vitro without light activation. *Exp Eye Res*. 2014; 124: 67–73. 24837142 10.1016/j.exer.2014.04.011 PMC4135181

[27] Mascharak S, desJardins-Park HE, Davitt MF, et al. Preventing Engrailed-1 activation in fibroblasts yields wound regeneration without scarring. *Science*. 2021; 372, doi:10.1126/science.aba2374. PMC9008875 33888614

[28] Livak KJ, Schmittgen TD. Analysis of relative gene expression data using real-time quantitative PCR and the 2−ΔΔCT method. *Methods*. 2001; 25: 402–408. 11846609 10.1006/meth.2001.1262

[29] Lipowsky HH, Kovalcheck S, Zweifach BW. The distribution of blood rheological parameters in the microvasculature of cat mesentery. *Circ Res*. 1978; 43: 738–749. 709740 10.1161/01.res.43.5.738

[30] Aragona M, Panciera T, Manfrin A, et al. A mechanical checkpoint controls multicellular growth through YAP/TAZ regulation by actin-processing factors. *Cell*. 2013; 154: 1047–1059. 23954413 10.1016/j.cell.2013.07.042

[31] Nardone G, Oliver-De La Cruz J, Vrbsky J, et al. YAP regulates cell mechanics by controlling focal adhesion assembly. *Nat Commun*. 2017; 8: 15321. 28504269 10.1038/ncomms15321 PMC5440673

[32] Wada K-I, Itoga K, Okano T, Yonemura S, Sasaki H. Hippo pathway regulation by cell morphology and stress fibers. *Development*. 2011; 138: 3907–3914. 21831922 10.1242/dev.070987

[33] Cai X, Wang K-C, Meng Z. Mechanoregulation of YAP and TAZ in cellular homeostasis and disease progression. *Front Cell Dev Biol*. 2021; 9: 673599. 34109179 10.3389/fcell.2021.673599 PMC8182050

[34] Dasgupta I, McCollum D. Control of cellular responses to mechanical cues through YAP/TAZ regulation. *J Biol Chem*. 2019; 294: 17693–17706. 31594864 10.1074/jbc.REV119.007963 PMC6873206

[35] Chan SW, Lim CJ, Chong YF, Pobbati AV, Huang C, Hong W. Hippo pathway-independent restriction of TAZ and YAP by angiomotin. *J Biol Chem*. 2011; 286: 7018–7026. 21224387 10.1074/jbc.C110.212621 PMC3044958

[36] Wang W, Huang J, Chen J. Angiomotin-like proteins associate with and negatively regulate YAP1. *J Biol Chem*. 2011; 286: 4364–4370. 21187284 10.1074/jbc.C110.205401 PMC3039387

[37] Leung CY, Zernicka-Goetz M. Angiomotin prevents pluripotent lineage differentiation in mouse embryos via Hippo pathway-dependent and -independent mechanisms. *Nat Commun*. 2013; 4: 2251. 23903990 10.1038/ncomms3251 PMC3741640

[38] Miller JW, Schmidt-Erfurth U, Sickenberg M, et al. Photodynamic therapy with verteporfin for choroidal neovascularization caused by age-related macular degeneration: results of a single treatment in a phase 1 and 2 study. *Arch Ophthalmol*. 1999; 117: 1161–1173. 10496388 10.1001/archopht.117.9.1161

[39] Nakajima H, Yamamoto K, Agarwala S, et al. Flow-dependent endothelial YAP regulation contributes to vessel maintenance. *Dev Cell*. 2017; 40: 523–536.e6. 28350986 10.1016/j.devcel.2017.02.019

[40] Discher DE, Janmey P, Wang Y-L. Tissue cells feel and respond to the stiffness of their substrate. *Science*. 2005; 310: 1139–1143. 16293750 10.1126/science.1116995

[41] Yeung T, Georges PC, Flanagan LA, et al. Effects of substrate stiffness on cell morphology, cytoskeletal structure, and adhesion. *Cell Motility*. 2005; 60: 24–34. 10.1002/cm.20041 15573414

[42] Chen W-S, Cao Z, Krishnan C, Panjwani N. Verteporfin without light stimulation inhibits YAP activation in trabecular meshwork cells: implications for glaucoma treatment. *Biochem Biophys Res Commun*. 2015; 466: 221–225. 26361148 10.1016/j.bbrc.2015.09.012

